# Activation of Biodefense System by Low-Dose Irradiation or Radon Inhalation and Its Applicable Possibility for Treatment of Diabetes and Hepatopathy

**DOI:** 10.5402/2012/292041

**Published:** 2012-02-09

**Authors:** Takahiro Kataoka, Kiyonori Yamaoka

**Affiliations:** Graduate School of Health Sciences, Okayama University, 5-1 Shikata-cho 2-chome, Kita-ku, Okayama-shi, Okayama 700-8558, Japan

## Abstract

Adequate oxygen stress induced by low-dose irradiation activates biodefense system, such as induction of the synthesis of superoxide dismutase (SOD) and glutathione peroxidase. We studied the possibility for alleviation of oxidative damage, such as diabetes and nonalcoholic liver disease. Results show that low-dose *γ*-irradiation increases SOD activity and protects against alloxan diabetes. Prior or post-low-dose X- or *γ*-irradiation increases antioxidative functions in livers and inhibits ferric nitrilotriacetate and carbon tetrachloride-induced (CCl_4_) hepatopathy. Moreover, radon inhalation also inhibits CCl_4_-induced hepatopathy. It is highly possible that low-dose irradiation including radon inhalation activates the biodefence systems and, therefore, contributes to preventing or reducing reactive oxygen species-related diabetes and nonalcoholic liver disease, which are thought to involve peroxidation.

## 1. Biological Response to Low-Dose Irradiation

Low-dose irradiation induces various stimulating effects, especially the activation of biological defense system such as antioxidative [1–9] and immune functions [9, 10]. For example, low-dose irradiation increases endogenous antioxidants in animal tissue. It has been reported that antioxidants such as superoxide dismutase (SOD) [3], glutathione peroxidase (GPx) [6], glutathione reductase (GR) [6, 7], glutathione [7], catalase [6], and thioredoxin [6] are activated and/or induced by low-dose irradiation. SOD changes superoxide anion (^•^O_2_) into hydrogen peroxide (H_2_O_2_), catalase, and GPx detoxify H_2_O_2_ into H_2_O and O_2_. Glutathione directly reacts with reactive oxygen species (ROS), and GPx catalyzes the destruction of H_2_O_2_ and hydroxyl radical (^•^OH). This catalysis generates oxidized glutathione (GSSG) and finally reduced glutathione (GSH) (total glutathione; t-GSH; GSH + GSSG). However, GR catalyzes the regeneration of GSH from GSSG. Thus GR and GPx are both the enzymes in the glutathione-regenerating pathway, and the changes of both activities are in a similar fashion. It is highly possible that low-dose X-irradiation activates the defensive systems in the living body and, therefore, contributes to preventing or reducing ROS-related injuries, which are thought to involve peroxidation.

Recently, we also demonstrated that radon inhalation activates antioxidative function in some organs. For example, radon inhalation activated antioxidative functions in the liver, kidney, lung, and brain of mice, including suggesting the possibility of a new therapy to treat liver, kidney, lung, and brain damage [11].

In this paper, we describe the activation of biodefense system by low-dose X- or *γ*-irradiation or radon inhalation and its applicable possibility for treatment of diabetes and nonalcoholic liver disease.

## 2. Protection against Alloxan Diabetes by Low-Dose **γ**-Irradiation before Alloxan Administration [12]

Diabetes is classified into insulin-dependent (Type I) and noninsulin-dependent (Type II) types. Alloxan selectively destroys insulin-secreting *β* cells in the islets of Langerhans in the pancreas, including sever glycosuria in experimental animals. Therefore, alloxan is one of the drugs used to analyze the developmental mechanism of Type I diabetes. The mechanism of the induction of diabetes by alloxan is speculated that oxidation-reduction induced by alloxan incorporated into *β* cells produces ROS such as ^•^O_2_, H_2_O_2_, and ^•^OH, which damage *β* cells [13, 14]. Therefore, we evaluated the protective effects of a single dose whole body ^60^Co *γ*-irradiation against alloxan-induced hyperglycemia in rats. Immediately before use the alloxan was dissolved in a solution of 50 mM citrate buffer (pH 4.5) and 0.6% NaCl and administered under ether anesthesia at 40 mg/kg *via* the daucal vein.

**Figure 1:**
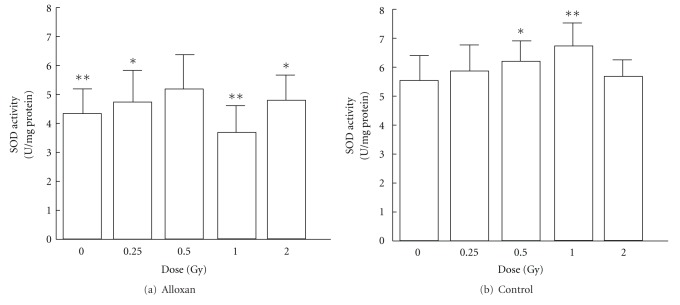
Changes in SOD activity in pancreas after alloxan administration and effects of low-dose *γ*-irradiation. Each value indicates the mean ± standard error of mean (SEM). *P < 0.05, **P < 0.01 versus sham-irradiated no-alloxan control group ((b). control, 0 Gy) [12].

**Figure 2:**
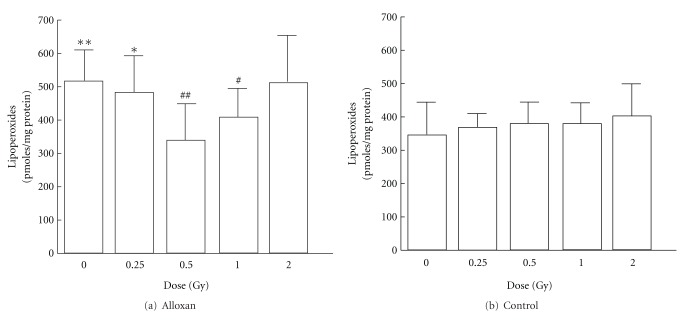
Changes in lipid peroxide levels in pancreas after alloxan administration and effects of low-dose *γ*-irradiation. Each value indicates the mean ± SEM. *P < 0.05, **P < 0.01 versus sham-irradiated no-alloxan control group. ^#^ *P* < 0.05, ^##^ *P* < 0.01 versus sham-irradiated alloxan control group [12].

**Figure 3:**
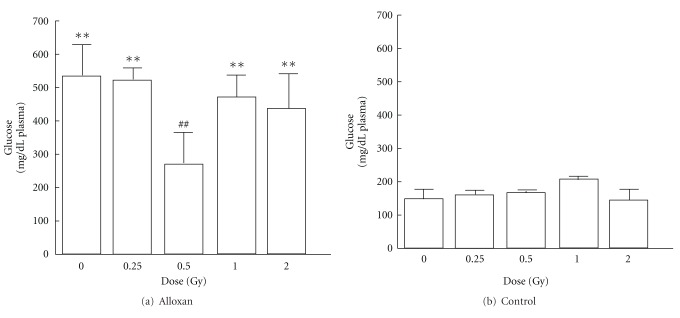
Changes in blood glucose levels after alloxan administration and effects of low-dose *γ*-irradiation. Each value indicates the mean ± SEM. **P < 0.01 versus sham-irradiated no-alloxan control group (B. control, 0 Gy). ^##^ *P* < 0.01 versus sham-irradiated alloxan control group [12].

In the control groups not treated with alloxan, the SOD activities in pancreas significantly increase in the 0.5 or 1.0 Gy irradiation groups compared with sham-irradiated control group. The alloxan-treated groups except 0.5 Gy-irradiated group showed a significant decrease in SOD activity compared with sham-irradiated control group (Figure 1). No significant irradiation dose dependence was observed in the lipid peroxide levels in pancreas of the control group not treated with alloxan. Among the alloxan-treated groups, the nonirradiation group showed a significant increase, but the 0.5 or 1.0 Gy irradiation groups showed a significant decrease, compared with the alloxan-treated-non-irradiation groups, to a level similar to that in the untreated group (Figure 2). In the control groups not treated with alloxan, no effects of the irradiation dose on the blood glucose level were observed. The alloxan-treated-irradiation groups except 0.5 Gy-irradiated group showed a significant increase in blood glucose level. A significant decrease in blood glucose level was observed in the alloxan-treated-0.5 Gy irradiation group compared with alloxan-treated-non-irradiation group, showing a level similar to that in the untreated-non-irradiation group (Figure 3).

The results of SOD and lipid peroxide level suggest that low-dose *γ*-irradiation increase SOD activity decreases lipid peroxide levels and protects against alloxan diabetes.

## 3. Prevention of Type I Diabetes by Low-Dose **γ**-Irradiation in NOD Mice [18]

Low-dose irradiation protects oxidative damage in animal tissue. Oxidative damage is known to be a major cause of many human diseases, such as diabetes. This study demonstrated the effects of low-dose irradiation on the progression of type I diabetes in mice. Nonobese diabetic (NOD) mice were treated with *γ*-irradiation, and the progression of the disease was monitored. About 60% of the control mice were positive for diabetes by 22 weeks of age, whereas only 10, 30, and 40% of the mice irradiated at 13, 12, and 14 weeks of age, respectively, presented a positive response. The insulin level of mice irradiated at 13 weeks of age was 89% higher than that of control mice. Many positively stained apoptotic cells were observed in the pancreas of control mice, whereas only a few were detected in the pancreas of irradiated mice at 13 weeks of age. In addition, SOD activity in pancreas of mice irradiated at 13 weeks of age was significantly higher than that of control mice. These findings suggest that activation of antioxidative function induced by low-dose irradiation suppresses progression of type I diabetes in NOD mice.

## 4. Effects of Radon Inhalation on Diabetes-Associated Substances [15]

A hot spring in Misasa-cho in Tottori Prefecture in Japan (Misasa hot spring) is famous as a radon hot spring. Indications for treatment at the Misasa hot spring, a radon producing radioactive spring, include diabetes. To clarify its mechanisms of action on these conditions, we examined the diabetes-associated substances in rabbits after radon inhalation. Radon water was (form Ikeda mineral springs at 4°C) used as radon source. The radon concentration of the water was approximately 14–18 kBq/l (high concentration group) and 7–10 kBq/l (low concentration group). Radon inhalation was maintained with an ultrasonic nebulizer using radon water.

**Figure 4:**
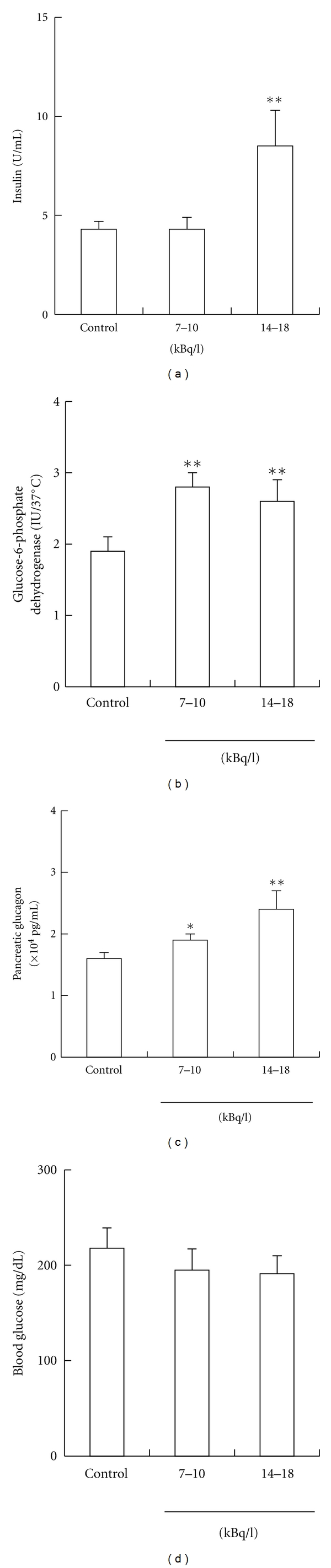
Dynamic changes in diabetes-associated substances of rabbit blood by radon inhalation. Each value indicates the mean ± SEM. The number of rabbits per experiment was ten for control, eight at 7–10 kBq/l, and nine at 14–18 kBq/l. Significance: *P < 0.05, **P < 0.01 versus control [15].

The results show that insulin level of high radon concentration group was significantly higher than that of control group. Glucose-6-phosphate dehydrogenase levels of low and high radon concentration groups were significantly higher than that of control group. Pancreatic glucagon levels of low or high radon concentration group were significantly higher than that of control group. However, no significant changes were observed in blood glucose levels (Figure 4).

These findings suggest that radon inhalation may contribute to the mechanism underlying alleviation of diabetic symptoms. In the future, clarification in detail of the mechanisms of these phenomena will be helpful toward understanding the effects on the functions of the living body or radon inhalation at the hot spring.

## 5. Inhibitory Effects of Prior Low-Dose X-Irradiation on Ferric-Nitrilotriacetate-Induced Hepatopathy in Rats [16]

Transient hepatopathy after ferric nitrilotriacetate (Fe^3+^-NTA) administration induced free radicals. This hepatopathy resembles excessive iron disease in humans. Therefore, this study demonstrated whether or not prior low-dose X-irradiation would suppress transient hepatopathy in rats. Rats were irradiated with a single dose of 0.5 Gy of X-ray. A sham producer without X-irradiation (sham irradiation) was performed on control rats. Two hours after X-irradiation, a single dose of Fe^3+^-NTA was administrated to rats.

**Figure 5:**
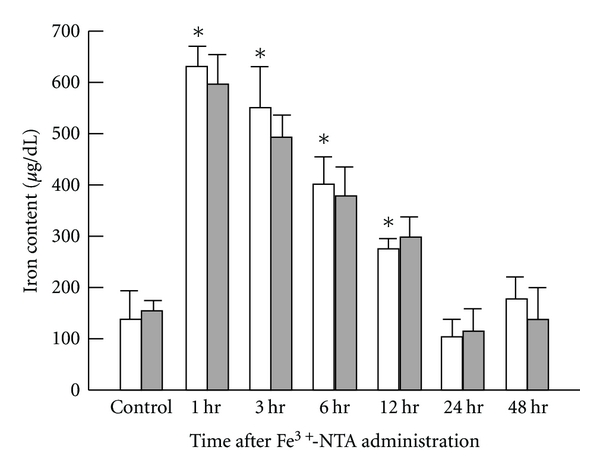
Time-dependent changes in the serum iron levels of Wister rats after Fe^3+^-NTA administration following low-dose X-irradiation. Each value indicates the mean ± SEM. The number of rats per experimental point is 10–12. *P < 0.05 by *t*-test, each Fe^3+^-NTA-administrated group value versus non-administrated control group value [16].

**Figure 6:**
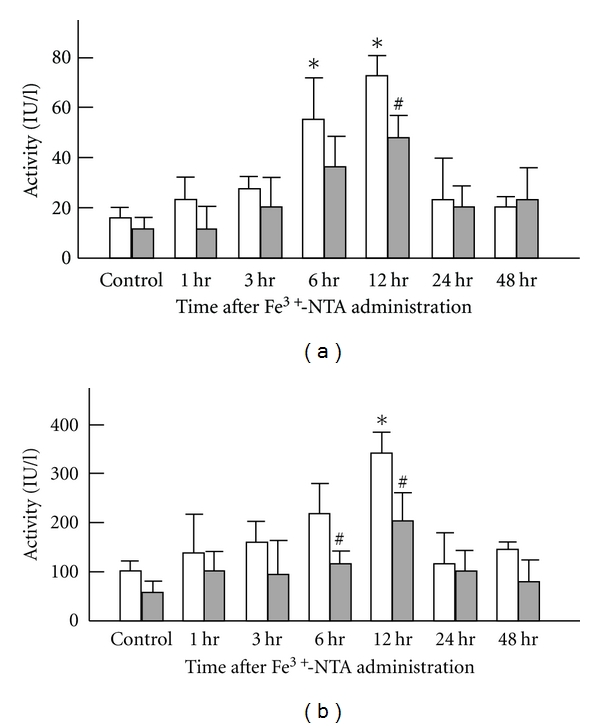
Time-dependent changes of the GPT and GOT activities in serum of Wister rats after Fe^3+^-NTA administration following low-dose X-irradiation. Each value indicates the mean ± SEM. The number of rats per experimental point is 10–12. *P < 0.05 by *t*-test, each Fe^3+^-NTA-administrated group value versus non-administrated control group value. ^#^ *P* < 0.05 by *t*-test, each group value at various intervals after irradiation versus the value at the same intervals after sham-irradiation [16].

**Figure 7:**
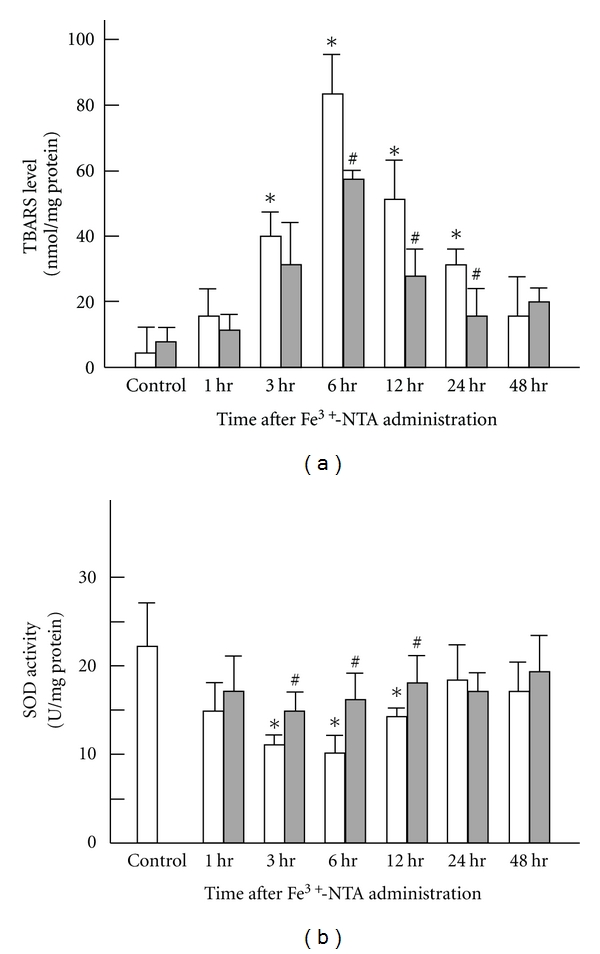
Time-dependent changes of the lipid peroxide levels and SOD activities in livers of Wister rats after Fe^3+^-NTA administration following low-dose X-irradiation. Each value indicates the mean ± SEM. The number of rats per experimental point is 10–12. *P < 0.05 by *t*-test, each Fe^3+^-NTA administrated group value versus non-administrated control group value. ^#^ *P* < 0.05 by *t*-test, each group value at various intervals after irradiation versus the value at the same intervals after sham irradiation [16].

At 1, 3, 6, and 12 hr after Fe^3+^-NTA administration, iron levels in serum of sham-irradiated groups were significantly increased compared with that of control level. No significant differences in iron level were observed between sham irradiation and 0.5 Gy irradiation (Figure 5). This result may indicate that there are no differences of the amount of free radicals induced by Fe^3+^-NTA administration between sham irradiation and 0.5 Gy irradiation. The glutamic oxaloacetate transaminase (GOT) activities of Fe^3+^-NTA-administrated rats were significantly increased at 12 hr, and the glutamic pyruvic transaminase (GPT) activity was significantly increased at 6 or 12 hr. However, at 12 hr after Fe^3+^-NTA administration, the GPT activity in serum of 0.5 Gy-irradiated rat was significantly lower than that of sham-irradiated rat, and at 6 or 12 hr the GOT activities in serum of 0.5 Gy-irradiated rat were significantly lower than that of sham-irradiated rat (Figure 6). Moreover, the thiobarbituric acid reacting substances (TBARSs), which show the level of oxidant injury, in liver of Fe^3+^-NTA-administrated rats were significantly increased at 3, 6, 12, or 24 hr compared with that of nonadministrated control rats. However, the increases in the lipid peroxide levels were suppressed by X-irradiation between 6 and 24 hr (Figure 7). These findings suggest that low-dose irradiation inhibits Fe^3+^-NTA-induced hepatopathy regardless of iron levels in serum.

To clarify the mechanism of the inhibitory effect, SOD activity, which is an antioxidant enzyme, was examined. The SOD activity in livers of sham-irradiated groups significantly decreased compared with that before administration. However, the decreases in the SOD activity were suppressed by X-irradiation between 3 and 12 hr (Figure 7). Based on these results, it is speculated that antioxidative function of the liver was activated by low-dose X-irradiation, resulting in the inhibition of Fe^3+^-NTA-induced hepatopathy.

## 6. Inhibitory Effects of Post-Low-Dose **γ**-Irradiation on Ferric-Nitrilotriacetate-Induced Mice Liver Damage [20]

We previously investigated whether or not X-irradiation at a dose of 0.5 Gy before Fe^3+^-NTA administration suppressed transient hepatopathy in rats. It is of interest whether 0.5 Gy *γ*-irradiation after Fe^3+^-NTA affects the oxidative liver damage. In this study, we investigated whether active oxygen-related diseases can be treated with low-dose irradiation, 0.5 Gy *γ*-irradiation to mice with Fe^3+^-NTA-induced transient hepatopathy.

Three hours after Fe^3+^-NTA administration, the activities of GOT and GPT in serum of the sham-irradiated and 0.5 Gy-irradiated groups reached a peak of about 3-4-fold that before administration. In the irradiated groups, increases in the activities were significantly suppressed between 24 and 48 hr after irradiation and returned to normal values earlier than those in the sham-irradiated group. Within 48 hr after irradiation, the lipid peroxide (malondialdehyde (MDA)) levels in the livers of the sham-irradiated groups significantly increased to about 4–7-fold that before Fe^3+^-NTA administration. In the irradiated groups, the relative increase in the lipid peroxide levels was smaller than those in the sham-irradiated groups, but both groups showed a similar time course. The increases in the lipid peroxide levels were significantly suppressed between 6 and 48 hr after irradiation, and each point value was significantly lower than the sham-irradiated group. Within 48 hr after irradiation, the t-GSH content in the livers of the sham-irradiated groups significantly decreased compared with that before Fe^3+^-NTA administration. In the irradiated group, the relative decrease in the t-GSH content was smaller than that in the sham-irradiated group, and both groups showed a similar time course. The decrease in the t-GSH content was significantly suppressed between 6 and 48 hr after irradiation.

Low-dose irradiation accelerated the rate of the recovery. The decrease in the transaminase activities and lipid peroxide levels showed that hepatopathy was recovered at 24–48 hr after irradiation. This may be because of the enhancement of antioxidant agents such as t-GSH by low-dose irradiation. These findings suggest that posttreatment with low dose of *γ*-ray is useful for clinical prevention and/or therapy of various ROS-related diseases.

## 7. Low-Dose **γ**-Irradiation Reduces Oxidative Damage Induced by CCl_4_ in Mouse Liver [21]

Carbon tetrachloride (CCl_4_) is frequently used as a chemical inducer of experimental liver damage. Transient hepatocellular disorder induced by CCl_4_ administration is thought to be induced by trichloromethyl radical trichloromethyl peroxy radical [22, 23]. Overproduction of these radicals initiates lipid peroxidation of polyunsaturated fatty acid in membrane and eventually leads to cell necrosis. These radicals induce an adverse reaction by forming radicals after its administration in the early stage between intracellular uptake and transformation into storage types. We believe that the clarification of glutathione-associated metabolism and neogenesis after irradiation is important for advancing studies of the mechanism of radio adaptive response. Therefore, we examined the effects of irradiation (0.5 Gy of *γ*-ray) reducing the oxidative damage in CCl_4_-hepatopathy mice. The irradiation of *γ*-rays was initiated 24 hr after the injection of CCl_4_.

The irradiation was found to accelerate the recovery from hepatopathy induced by CCl_4_. Fatty degeneration was observed in CCl_4_ administration group. However, low-dose irradiation inhibits fatty degeneration. The lipid peroxide levels in livers were greatly elevated by CCl_4_ treatment. However, those levels decreased more rapidly after the irradiation. GOT and GPT activities were remarkably increased by CCl_4_ injection, and those of low-dose irradiation group rapidly returned to normal values compared with the sham irradiation group.

The t-GSH content of low-dose irradiation group was higher than that of the sham irradiation group. The irradiation group showed accelerated recovery of the t-GSH content which had decreased after CCl_4_ administration.

GR activity obviously decreased with CCl_4_ administration. This lowered GR activity rapidly elevated to normal level with 0.5 Gy irradiation in 3 hr. In contrast, the sham irradiation group did not show any change in the activity 3 hr after the irradiation.

These findings suggest that low-dose irradiation relieved functional disorder at least in the liver of mice with active oxygen diseases.

## 8. Inhibitory Effects of Prior or Post-Low-Dose X-Irradiation on Carbon Tetrachloride-Induced Hepatopathy in Acatalasemic Mice [17, 19]

Catalase is an important component of the cellular defense system against damage induced by ROS. In terms of the regulation of intracellular H_2_O_2_ in biological systems, both catalase and GPx are responsible; the former was suggested to play a major role in H_2_O_2_ breakdown, particularly when H_2_O_2_ is overproduced [24].

**Figure 8:**
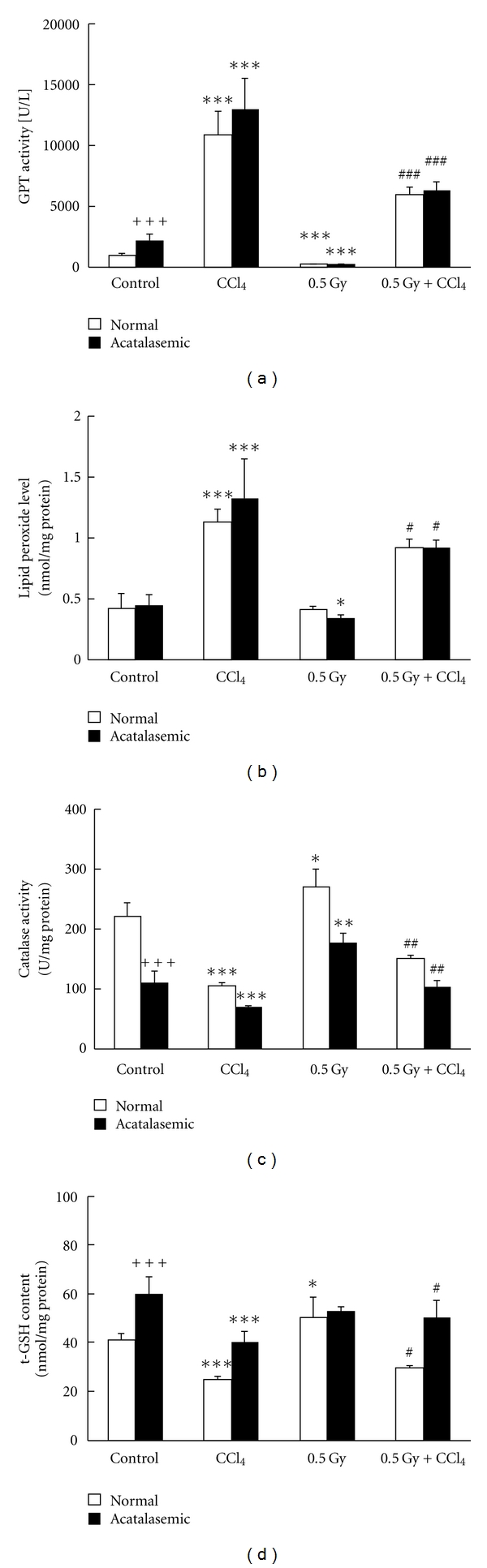
Differences in the GPT activity in serum, lipid peroxide level, catalase activity, and t-GSH content in liver between normal and acatalasemic mice, six to seven weeks of age, under nontreated control, CCl_4_ administration, 0.5 Gy X-irradiation, or 0.5 Gy-irradiation prior to CCl_4_ administration. Each value indicates the mean ± SEM. The number of mice per experimental point is 6–12. ^+++^ *P* < 0.001 by *t*-test, acatalasemic mice value versus normal mice value under no treatment. *P < 0.05, **P < 0.01, ***P < 0.001 by *t*-test, each normal mice value or acatalasemic mice value under CCl_4_ administration or 0.5 Gy X-irradiation versus under no treatment, respectively. ^#^ *P* < 0.05, ^##^ *P* < 0.01, ^###^ *P* < 0.001 by *t*-test, each acatalasemic mice value or normal mice value under CCl_4_ administration after 0.5 Gy X-irradiation versus under CCl_4_ administration [17].

**Figure 9:**
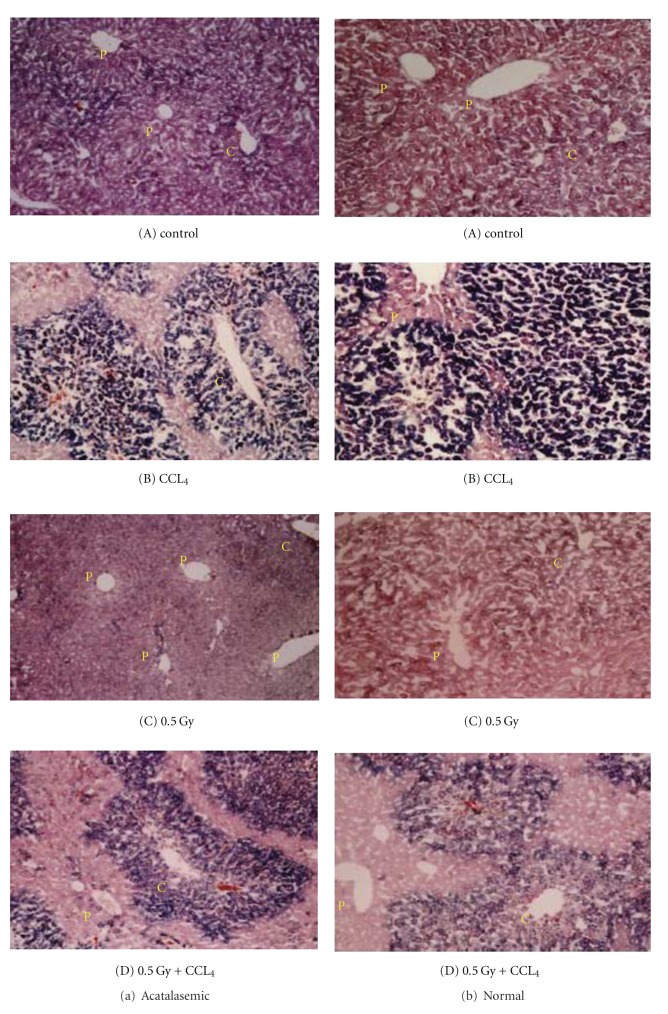
Differences in liver histopathology between acatalasemic (a) and normal (b) mice under non-treated control (A) CCl_4_ administration (B), 0.5 Gy X-irradiation (C), or 0.5 Gy irradiation prior to CCl_4_ administration (D). All figures under lower magnification (X 100) are stained with Sudan Black B (black-colored) for the detection of fatty degeneration surrounding the central vein (C) and the portal vein (P). The areas of cell necrosis surrounding the central vein in the CCl_4_-administrated mice after 0.5 Gy X-irradaiation were smaller in both mice in comparison with the CCl_4_-administrated mice. No obvious difference was noted in the extent of the centrilobular hepatocyte damage by nontreatment or 0.5 Gy X-irradiation in both mice [17].

Hypoacatalasemic mouse has higher catalase activity than acatalasemic mouse and lower catalase activity than normal mouse. For example, the catalase activities of blood and tissues in acatalasemic mouse (C3H/AnLCs^b^Cs^b^) are one-tenth to half, and those of hypoacatalasemic mouse (C3H/AnLCs^c^Cs^c^) are two-thirds those of normal mouse (C3H/AnLCs^a^Cs^a^), respectively [25]. The catalase activities in blood and organs of the acatalasemic (C3H/AnLCs^b^Cs^b^) mouse of C3H strain are lower than those of the normal (C3H/AnLCs^a^Cs^a^) mouse. It was reported that CCl_4_-induced hepatotoxicity was enhanced in acatalasemic mice in comparison with the normal mice in the later phases of liver injury [26]. This might be due to the increased formation of ^•^OH in the absence of catalase instead of ^•^O_2_. Therefore, we examined the effects of prior or post-low-dose X-irradiation, which reduced the oxidative damage under CCl_4_-induced hepatopathy in the acatalasemic or normal mice. The acatalasemic mice showed a significantly lower catalase activity and a significantly higher GPx activity compared with those in the normal mice. Moreover, low-dose irradiation increased the catalase activity in the acatalasemic mouse liver to a level similar to that of the normal mouse liver. Analyses of blood GOT and GPT activity and lipid peroxide levels showed that CCl_4_-induced hepatopathy was inhibited by low-dose irradiation (Figure 8). Other results in this report show that histological examinations of liver tissues revealed no significant changes before CCl_4_ administration in acatalasemic and normal mice from either the prior low-dose-irradiated or the control sham-irradiated groups. Liver tissues exhibited that fatty degeneration was more extensive in acatalasemic mouse liver than in the normal mice. However, in low-dose irradiated and CCl_4_-treated groups, there was no obvious difference in the extent of the fatty degeneration between the acatalasemic and normal mice (Figure 9). These findings may indicate that the free radical reaction induced by the lack of catalase and the administration of CCl_4_ is more properly neutralized by high GPx activity and low-dose irradiation in the acatalasemic mouse liver.

Next, we examined the effects of post-low-dose X-irradiation on CCl_4_-induced acatalasemic mice liver damage. The 0.5 Gy irradiation after CCl_4_ administration decreased the activities of GOT and GPT in the acatalasemic or normal mice blood compared with those of CCl_4_-treated mice. However, high-dose irradiation (15 Gy) after CCl_4_ administration increased the GOT activities in the acatalasemic or normal mice blood compared with those of CCl_4_-treated mice (Table 1). CCl_4_ administration significantly increased the lipid peroxide levels in livers of acatalasemic or normal mice. However, the 0.5 Gy irradiation after CCl_4_ administration significantly decreased the lipid peroxide levels in livers of normal mice. CCl_4_ administration significantly increased the activities of SOD and catalase in livers of acatalasemic or normal mice. However, the 0.5 Gy irradiation after CCl_4_ administration significantly increased the SOD activity in livers of acatalasemic mice and catalase activities in livers of acatalasemic or normal mice (Table 2). In addition, other results in this report showed that pathological disorder was improved by 0.5 Gy irradiation. The fat degeneration in normal mice was quickly reduced, in contrast to acatalasemic mice. These findings suggest that low-dose irradiation after CCl_4_ administration accelerates the rate of recovery and that catalase plays an important role in the recovery from hepatopathy induced by CCl_4_, in contrast to high-dose irradiation.

## 9. Radon Inhalation Protects Mice from Carbon-Tetrachloride-Induced Hepatic and Renal Damage [27]

We assessed whether radon inhalation provided protection from CCl_4_-induced hepatic and renal damage in mice. Mice were subjected to intraperitoneal injection of CCl_4_ after inhaling approximately 18 kBq/m^3^ radon for 6 hr. Radon inhalation significantly increased t-GSH content and GPx activity in the liver and kidney. Injection of CCl_4_ was associated with significantly higher levels of GOT and alkaline phosphatase (ALP) activity in serum, and pretreatment with radon significantly decreased the GOT and ALP activity associated with CCl_4_ injection, suggesting that radon inhalation alleviates CCl_4_-induced hepatopathy. These findings suggested that radon inhalation activated antioxidative functions and inhibited CCl_4_-induced hepatopathy.

## 10. Conclusion

It is highly possible that low-dose irradiation including radon inhalation activates the biodefence systems and, therefore, contributes to preventing or reducing reactive oxygen species-related diabetes and nonalcoholic liver disease, which are thought to involve peroxidation.

## Figures and Tables

**Table 1:** Temporal changes in aminotransferases in blood of acatalasemic or normal mice at each sham, 0.5 Gy, or 15 Gy X-ray irradiation following CCl_4_ administration. Each value indicates the mean ± SEM. The number of mice experimental points is 5. ^+++^ *P* < 0.001 versus normal mouse *P < 0.05, **P < 0.01 versus control [19].

		Mice	
	Treatment	Normal	Acatalasemic
GOT activity [KU/L]	Control	0.84 ± 0.09	2.20 ± 0.15^+++^
	CCl4 + Sham	5.62 ± 0.47	7.74 ± 0.56
	CCl4 + 0.5 Gy	4.57 ± 0.43*	6.61 ± 0.51*
	CCl4 + 15 Gy	7.52 ± 0.55*	9.65 ± 0.62*

GPT activity [KU/L]	Control	0.97 ± 0.10	2.17 ± 0.15^+++^
	CCl4 + Sham	7.36 ± 0.54	9.14 ± 0.60
	CCl4 + 0.5 Gy	5.27 ± 0.46**	7.62 ± 0.55**
	CCl4 + 15 Gy	7.98 ± 0.55	10.14 ± 0.64*

**Table 2:** Temporal changes in antioxidant-associated substances in liver of acatalasemic or normal mice at each sham, 0.5 Gy, or 15 Gy X-ray Irradiation following CCl_4_ administration. Each value indicates the mean ± SEM. The number of mice experimental points is 5. ^+++^ *P* < 0.001 versus normal mouse *P < 0.05, ***P < 0.001 versus control, ^#^ *P* < 0.05, ^###^ *P* < 0.001, versus *CCl*_4_ + Sham [19].

		Mice	
	Treatment	Normal	Acatalasemic
Lipid peroxide level [nmol/mg protein]	Control	0.39 ± 0.06	0.49 ± 0.08
	CCl4 + sham	1.04 ± 0.20***	1.5 ± 0.26***
	CCl4 + 0.5 Gy	0.80 ± 0.08^#^	1.28 ± 0.18
	CCl4 + 15 Gy	1.01 ± 0.12	1.17 ± 0.2

SOD activity [U/mg protein]	Control	48.6 ± 3.8	55.5 ± 3.7
	CCl4 + sham	20.7 ± 5.0***	20.4 ± 3.9***
	CCl4 + 0.5 Gy	22.0 ± 2.4	29.1 ± 6.4^#^
	CCl4 + 15 Gy	15.2 ± 2.4	20.7 ± 3.4

Catalase activity [U/mg protein]	Control	250 ± 25.0	95 ± 14.5^+++^
	CCl4 + sham	107 ± 23.2***	68 ± 13.3*
	CCl4 + 0.5 Gy	205 ± 14.1^###^	110 ± 10.0^###^
	CCl4 + 15 Gy	167 ± 30.9^#^	53 ± 11
